# The Effect and Action Mechanisms of Oligochitosan on Control of Stem Dry Rot of *Zanthoxylum* *bungeanum*

**DOI:** 10.3390/ijms17071044

**Published:** 2016-06-30

**Authors:** Peiqin Li, Zhimin Cao, Zhou Wu, Xing Wang, Xiuhong Li

**Affiliations:** 1 Department of Forest Protection, College of Forestry, Northwest A&F University, Yangling 712100, China; zmcao@nwsuaf.edu.cn (Z.C.); 15229371653@163.com (Z.W.); 2 Department of Chemistry and Chemical Biology & the Center for Biotechnology and Interdisciplinary Studies, Rensselaer Polytechnic Institute, Troy, NY 12180, USA; wangx28@rpi.edu; 3 Department of Chemical Processing of Forest Products, College of Forestry, Northwest A&F University, Yangling 712100, China; lixiuhong@nwsuaf.edu.cn

**Keywords:** oligochitosan, dry rot, *Fusariumsambucinum*, *Zanthoxylumbungeanum*, defensive enzyme, phenolics

## Abstract

In this report, the effects of two oligochitosans, i.e., oligochitosan A (OCHA) and oligochitosan B (OCHB), on control of dry rot of *Zanthoxylum* *bungeanum* (*Z. bungeanum*) caused by *Fusarium* *sambucinum* (*F. sambucinum*) were evaluated. First, both oligochitosans show desirable ability to decrease the infection of *F. sambucinum*. Second, the oligochitosans strongly inhibit the radial colony and submerged biomass growth of *F. sambucinum*. Lastly, these oligochitosans are capable of increasing the activities of phenylalanine ammonia lyase (PAL), polyphenoloxidase (PPO) and peroxidase (POD) significantly, as well as enhancing the content of total phenolics in *Z. bungeanum* stems. These findings indicate that the protective effects of OCHA and OCHB on *Z. bungeanum* stems against dry rot may be associated with the direct fungitoxic function against pathogen and the elicitation of biochemical defensive responses in *Z. bungeanum* stems. The outcome of this report suggests that oligochitosans may serve as a promising natural fungicide to substitute, at least partially, for synthetic fungicides in the disease management of *Z. bungeanum*.

## 1. Introduction

*Zanthoxylum* *bungeanum* (*Z. bungeanum*), named Huajiao in Chinese, is an aromatic plant of Rutaceae*.* It is a native shrub or small tree that originated from the southwestern part of China [1]. Since it is widely used as a pungent ingredient and seasoning in many East Asian countries, planting of *Z. bungeanum* has become an important estate for farmers in China for its economic value [2]. It has been reported that *Z. bungeanum* is frequently damaged by different kinds of plant diseases in its growth, especially during the process of stem dry rot [3], which, caused by *Fusarium* *sambucinum* (*F. sambucinum*), can seriously affect growth of *Z. bungeanum* and therefore result in a significant decrease of its yield [4]. Up to now, the main measures for controlling *Z. bungeanum*’s diseases and pests rely on chemically synthesized pesticides [5]. Synthetic pesticides have their own advantage, but the side effect of indiscriminate use of these pesticides on crops has aroused increasing concerns for pesticide residue, pathogenic resistance to pesticide, and resurgence of pests [6]. Hence, there are great demands for nontoxic but effective alternatives to current synthetic pesticides, aiming for reducing the incidence of plant disease and minimizing the negative effect of synthetic pesticides on human and environmental health [7].

As the potential substitutes for current synthetic pesticides, the natural products derived from animal and plants have attracted growing attention of plant pathologists because of their biodegradable nature and antimicrobial activity compatible with the synthetic pesticides [8]. Oligochitosan, with a chemical structure of β-(1→4)-*N*-acetyl-d-glucosamine, is one type of such biodegradable products that derive from crustaceous shells or fungal cell walls. It is a group of nontoxic and bioactive high molecular polymers with optimal water-solubility, which also shows the abilities to halt the growth of pathogens, to elicit marked morphological changes, structural alterations and molecular disorganization of the fungal cells, and to induce plant defense response [9]. Recent studies have well documented fungicidal activities of the oligochitosan both in vitro and in vivo [10], including the inhibition of radial and mycelia growth of plant pathogens such as *Fusarium* *solani* [11], *Puccini* *aarachidis* [12], *Alternaria alternate* [13], *Aspergillus* *niger* [14], and *Botrytis cinerea* [15], the suppression of formation and germination of fungal spore [14], the diminishing of the spore viability and germ tube growth [16], and the reduction of the plant disease incidence [17]. Moreover, oligochitosan also demonstrated the capabilities of inducing the defensive responses in plant tissues, including biosynthesis of phytoalexin [18], of increasing tyrosine ammonia lyase (TAL), phenylalanine ammonia lyase (PAL), polyphenoloxidase (PPO), peroxidase (POD), and chitinase activities [19,20], and of accumulating of phenolic compounds [21]. It was also discovered that the function of oligochitosan is not only influenced by its molecular weight (*M*w), degree of acetylation (DA) and polymerization (DP), but also related to the type of plants where the oligochitosan is applied. For example, obvious control effects of one oligochitosan with 5–10 kDa *M*w and 65% DA on wheat root rot were observed, while for the late blight disease of potato, the oligosaccharide with 2–6 KDa *M*w and 85% DA exhibited the best immunnomodulate activity of resistance [22,23,24]. Therefore, it is necessary to explore for the most effective oligochitosan to suit the control of corresponding plant diseases. Obtaining such oligochitosan will further accelerate the development of integrated disease management and sustainable agriculture.

As the planting of *Z. bungeanum* shows high economic value but no precedent work was reported of using oligochitosan to control the disease of *Z. bungeanum*, herein we evaluated the control effects of two kinds of oligochitosans (with different molecular weight and degrees of acetylation) on dry rot of *Z. bungeanum*, by determining their influence on radial colony and submerged biomass growth of *F.* *sambucinum* in vitro, and by analyzing the changes of defense-related physiological responses in *Z. bungeanum* tissues after oligochitosan treatment. The reported assay could help to lay the foundation for the nuisanceless prevention and management of dry rot of *Z. bungeanum*.

## 2. Results and Discussion

### 2.1. Protective Effects of Oligochitosan on the Stem of Z. bungeanum against Dry Rot

**Figure 1:**
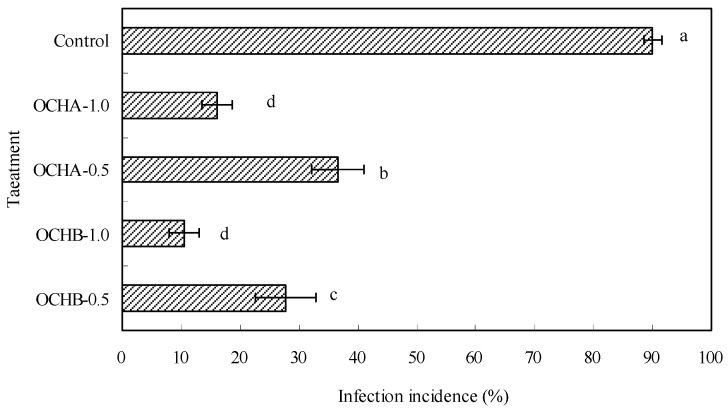
Protective effects of oligochitosan A (OCHA) and oligochitosan B (OCHB) on *Zanthoxylum* *bungeanum* (*Z. bungeanum*), stems against infection of *Fusarium* *sambucinum* (*F. sambucinum*). OCHA-1.0 and OCHA-0.5 represented concentrations of OCHA of 1.0 and 0.5 mg/mL, respectively. OCHB-1.0 and OCHB-0.5 represented concentrations of OCHB of 1.0 and 0.5 mg/mL, respectively. The error bars represented standard deviations of the means from three independent samples. Values followed by different letters (i.e., a, b, c and d) indicated significant differences among the different treatments at *p* = 0.05 level.

As illustrated in Figure 1, the protective effects of oligochitosan A (OCHA) and oligochitosan B (OCHB) on the stem of *Z. bungeanum* against dry rot infected by *F.* *sambucinum* were evaluated through the infection incidence. Infection incidence showed negative correlation with the protective effect of oligochitosan. The lower the infection incidence, the stronger protective effect of oligochitosan on plant against disease. This is consistent with the data reported previously by Yonni *et al.* [25]. The success in the dry rot infection was confirmed by chlorisis and browning of bark in the inoculated site. Compared with the infection incidence of control sample, all of the oligochitosan treatments have shown significant inhibition against infection. The best protective effect was achieved with 10.56% infection incidence when 1.0 mg/mL OCHB was applied. This is significantly lower than the effect of the control that had a 90% infection incidence. The same concentration of OCHA (1.0 mg/mL) showed a slight less protective effect (16.11% infection incidence) compared to OCHB. As predicted when the concentration of OCHA or OCHB was cut in half to 0.5 mg/mL, their inhibitory effects on the infection (with respective infection incidences as 36.67% and 27.78%) were less significant than the ones of the 1.0 mg/mL samples but much greater than that of the control. In short, the infection incidence study showed that both OCHA and OCHB exhibited strong protective effects on *Z. bungeanum* stems against infection of *F. sambucinum* and this effect is concentration dependent: the higher of the oligochitosan concentration, the stronger of the protective effect. Furthermore, OCHA and OCHB also showed different protective levels, which might be related to the differences of the two oligochitosans in component, molecular weight, and degree of acetylation, originated from different preparing approaches.

It has been well demonstrated that oligochitosans possess excellent protective effects on plants against many different diseases through inhibition of the RNA synthesis in some pathogenic fungi, the action of which reduces the viability of fungal cells, as well as fungal growth, by activating the defense response in plant cells [26]. In this study, oligochitosan might enter *Z. bungeanum* cells through puncture wounds, and then get accumulated in plant cells to induce resistance against *F.* *sambucinum* by turning on the defense response from corresponding entities [27]. Hence, a further investigation was carried out (as discussed below) to examine the effects of oligochitosan on the growth of *F. sambucinum* and the defensive physiological response of *Z. bungeanum*.

### 2.2. Effects of Oligochitosan A (OCHA) and Oligochitosan B (OCHB) on Radial and Submerged Growth of F. sambucinum

To understand the mechanisms of oligochitosan’s protection effect on *Z. bungeanum* stems against dry rot, the impacts of OCHA and OCHB on radial colony and submerged biomass growth of *F. sambucinum* were studied. Table 1 summarizes the inhibition effects of OCHA and OCHB on the growth of *F. sambucinum* by utilizing radial growth inhibition (RGI) and biomass growth inhibition (BGI). As presented in Table 1, both OCHA and OCHB directly inhibited radial colony and submerged biomass growth of *F. sambucinum* in all treatments with a positive correlation between the value of RGI or BGI and their respective inhibitory effect. These results were accordant with previous research on antifungal activities of oligochitosans [28,29]. The inhibition of *F. sambucinum* growth was affected by the concentration and form of oligochitosan. By calculating RGI and GBI at different time points, it was concluded that OCHA or OCHB shows a long inhibitory effective duration on growth of *F. sambucinum*. Even on the 10th day after the oligochitosan was introduced, the high values of RGI and BGI were still observed. In our study, we observed that the values of both RGI and BGI increased as the concentration of oligochitosan was increased from 0.2 to 1.0 mg/mL. More specifically, for OCHA the highest inhibitory effect was observed when its concentration reached at 1.0 mg/mL with 89.73% RGI and 84.64% BGI on the 10th day. Similarly for OCHB, the high was obtained when its concentration is 1.0 mg/mL with 95.69% RGI and 92.34% BGI on the 10th day. Generally, OCHB shows slightly stronger inhibition against growth of *F. sambucinum.*

The inhibitory effects generated from OCHA and OCHB might be resulted from their direct interaction with dry rot in *Z. bungeanum* stems. However, the mechanism behind the effect is still largely elusive. It has been postulated that oligochitosan may interfere with negatively charged residues of macromolecules located on the fungal cell surface, and the polycationic nature of oligochitosan will then lead to the leakage of proteinaceous constituents and intracellular electrolytes [30,31]. Moreover, there is also a possibility that the interaction of diffused hydrolysis products of oligochitosan with microbial DNA may in turn suppress the synthesis of mRNA and protein of fungi [26].

### 2.3. Elicitation of Phenylalanine Ammonia Lyase (PAL), Polyphenoloxidase (PPO) and Peroxidase (POD) Activities in Z. bungeanum by Oligochitosan

**Figure 2:**
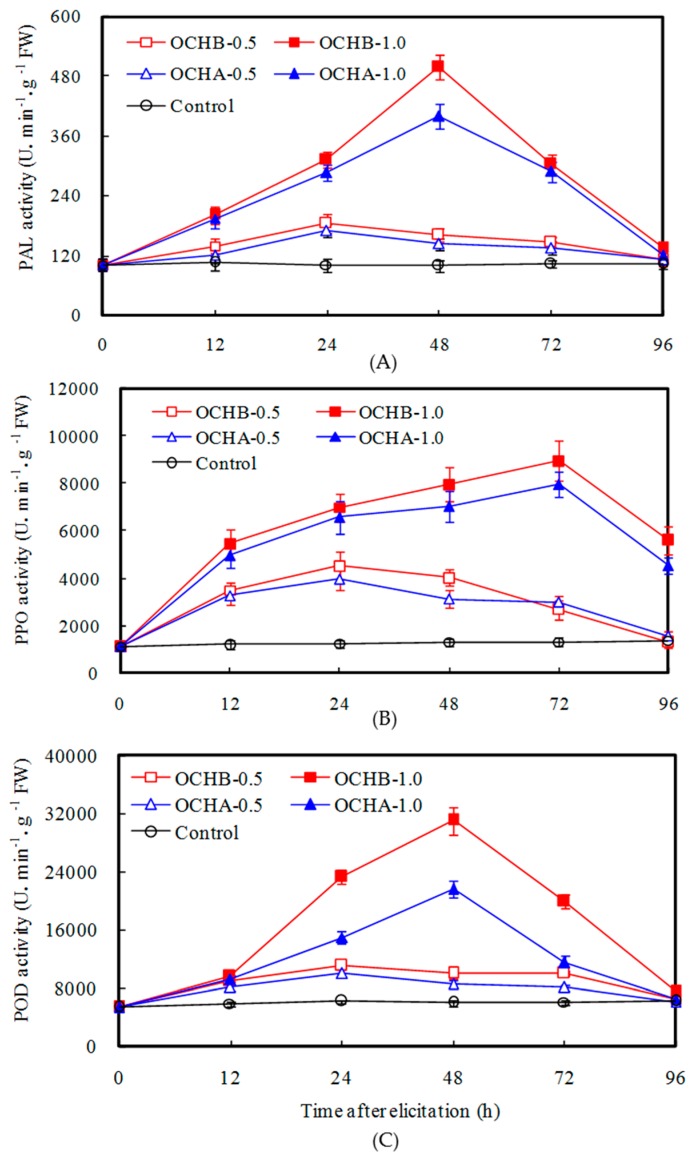
Effects of OCHA and OCHB on: PAL (**A**), PPO (**B**) and POD (**C**) activities in twigs of *Z. bungeanum*. The error bars represent standard deviations of the means from three independent samples; FW, fresh weight.

It was reported previously that oligochitosan exhibited desirable protective effects on plant diseases by inhibiting the growth of pathogenic fungi either directly or by inducing defensive response in plants [21]. In this study, we have carried out a systemic study of the OCHA and OCHB effects on elicitation of PAL, PPO and POD in *Z. bungeanum* twigs. As shown in Figure 2, both OCHA and OCHB have significantly induced the enhancements of PAL, PPO and POD activities. More specifically, OCHB exhibited a stronger inductive capability compared to OCHA with the same concentration. It was also demonstrated that higher activities of PAL, PPO and POD were induced by higher concentration of oligochitosan. As illustrated in Figure 2A, PAL activity reached the highest level 48 h after *Z. bungeanum* twig was treated with 1.0 mg/mL OCHB, which showed a 5.01-fold increase in activity compared to the control. Under the same elicitation condition for OCHA, the highest PAL activity was 4.01-fold increase, which was slightly lower than that of OCHB. Figure 2B shows that the highest PPO activity was induced by 1.0 mg/mL OCHB at 72 h, while for OCHA, the optimal elicitation condition for PPO was under the concentration of 1.0 mg/mL at 72 h with 6.01-fold PPO activity increase. From Figure 2C, the maximum POD activity was induced by 1.0 mg/mL OCHB at 72 h, which was 5.16-fold increase over the control.

In the present study, we have shown that PAL, PPO and POD activities in *Z. bungeanum* twig were significantly increased by the addition of OCHA or OCHB. As demonstrated before, PAL is a key enzyme catalyzing the phenylpropanoid metabolism, resulting in the biosynthesis of phenolic compounds that have antifungal activity [32]. PPO in plants can oxidize monophenol, diphenol or trihydric to their corresponding quinines, which can restrict the growth of the plant pathogen [33]. The increase of POD activity elicited by oligochitosan was also proven to be related to the increased resistance against pathogenic fungi [28]. POD participates in the cell reinforcement and is involved in the final steps of lignin biosynthesis by crosslinking cell-wall proteins [34]. When *Z. bungeanum* twigs were treated by oligochitosan, the increases of the PAL, PPO and POD activities may lead to the accumulation of total phenolics, the prompt oxidation of polyphenols, or the rapid biosynthesis of lignin to strengthen of structural barriers, all of which might be the factors that limit the activities of pathogen. Hence, we concluded that the protective effect of OCHA or OCHB on *Z. bungeanum* twig against *F. sambucinum* infection might be implicated in its induction ability of defense-related enzymes. This conclusion is consistent with the previous reports showing that oligochitosan has the potential to stimulate the enhancement of defense-related enzymes in other plants [7,21]. In addition, the differences of the PAL, PPO or POD activity induced by OCHA and OCHB were also evident, while OCHB displayed a better induction ability, which might be determined by its molecular weight, degree of polymerization and acetylation, and the chemical preparation approach for OCHB.

### 2.4. Effects of Oligochitosan on Content of Total Phenolics in Z. bungeanum

**Figure 3:**
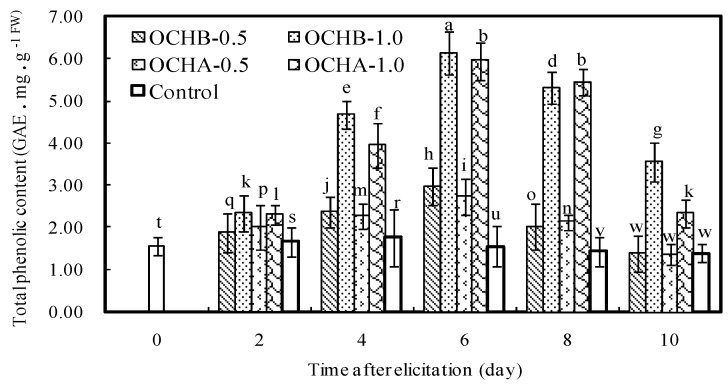
Effects of OCHA and OCHB on total phenolics in *Z. bungeanum* twig. The error bars represent standard deviations of the means from three independent samples. Values followed by different letters indicated significant differences among the different treatments at *p* = 0.05 level. FW means fresh weight of plant material.

Previous research has documented that oligochitosan possessed the capacity to stimulate the synthesis of phenolic compounds, which is one of defense-related secondary metabolites in plants [35]. Herein to test if OCHA and OCHB carry the similar functionality, we have quantified the effects of OCHA and OCHB on the content of total phenolics in *Z. bungeanum* and graphed the collated experimental data shown in Figure 3. Compared with the control, the amount of total phenolics after treated with oligochitosan was significantly increased, with a positive correlation between the concentration of oligochitosan and its elicitation ability. The amount of total phenolics reached the peak on the 6th day after the addition of 1.0 mg/mL OCHA (3.81-fold increase) or OCHB (3.94-fold increase). Figure 3 also shows that the amount of total phenolics in *Z. bungeanum* twig was slightly increased in the first two days after the elicitation started, and then was rapidly elevated from the 3rd day until the amount of phenolics reached the peak on the 6th day, before it began to decrease. As phenolic compounds in plants play a predominant role in antifungal activity [36], we concluded that the protective effect of OCHA or OCHB on *Z. bungeanum* stems against dry rot was partially attributed to the inductive accumulation of phenolic compound.

Oligochitosan has been considered as an admirable natural alternative to some chemical pesticides because of its biodegradable nature, antimicrobial activity and elicitation of defensive reactions in plants [37]. The present results show that OCHA and OCHB are extremely effective for controlling dry rot of *Z. bungeanum* stem. Overall, OCHB is more effective than OCHA on controlling dry rot of *Z. bungeanum*, inhibiting growth of *F. sambucinum*, enhancing the activities of PAL, PPO and POD, and stimulating the synthesis of total phenolics, which might be resulted from their different preparation methods that lead to the different molecular weight (*M*w), monosaccharide component, degree of acetylation (DA) and polymerization (DP) [38]. The mechanisms of oligochitosan on control of plant disease have been reported to be attributed to direct inhibition of the growth of pathogen, enhancement of defensive enzyme activities, and total phenolics in the host plant [20,21]. The molecular mechanism of oligochitosan inducing resistance of the plant host to its pathogen was also reported by gene expression, which might brighten our future research [39]. Hence, further research is urgently needed to find out molecular mechanism of OCHA or OCHB on control dry rot of *Z. bungeanum* and determine the specific relationship between structure and activity, which would establish the foundation for the appropriate application of oligochitosan for plant disease management.

In the present research, the oligosaccharide we employed could be perfectly dissolved in water, which would be very helpful for the utilization of oligochitosan as bio-pesticide on crop protection. The methods we used to study the control effects of oligochitosan on dry rot of *Z. bungeanum* were by immersing the cut twigs into oligochitosan solution or wrapping the inoculated sites with cotton absorbing oligochitosan solution. It demonstrates that oligochitosan could be absorbed by plant stems through cut wound or lenticels. In vivo inhibition detection model also proves oligochitosan could inhibit the growth of pathogen of dry rot directly. It might be concluded when using oligochitosan to control dry rot of *Z. bungeanum* in the field, direct spraying or injection of oligosaccharide solution into the host plant or wrapping disease spot using oligochitosan solution cotton would be effective. However, the exploitation of oligochitosan bio-pesticide formulation is also important for its practical application. Furthermore, considering high price of pure oligochitosan and the large application dosage of oligochitosan in control of plant diseases, the oligochitosan in our present research were mixtures not pure compounds, but their lower purchase price and good efficacy on control of dry rot of *Z. bungeanum* would be extremely possible to be applied on a large-scale field.

## 3. Materials and Methods

### 3.1. Chemicals

Oligochitosan A (OCHA) and oligochitosan B (OCHB) were purchased from Qindao BZ-Oligo Biotech Co., Ltd., (Qiandao, China). According to product descriptions, the OCHA was the hydrolyzate mixture of chitosan by chlorhydric acid with a molecular weight (*M*w) <1500 Da and a degree of acetylation (DA) higher than 90%, while OCHB was the hydrolyzate mixture of chitosan by enzyme with molecular weight <3000 Da and a degree of acetylation (DA) lower than 90%. Fourier transform infrared spectroscopy (FT-IR) and electrospray ionization mass spectrometry (ESI-MS) analyses of OCHA and OCHB were carried out. All the other chemicals were purchased from JieCheng Chemical and Glass Company (Yangling, China).

**Figure 4:**
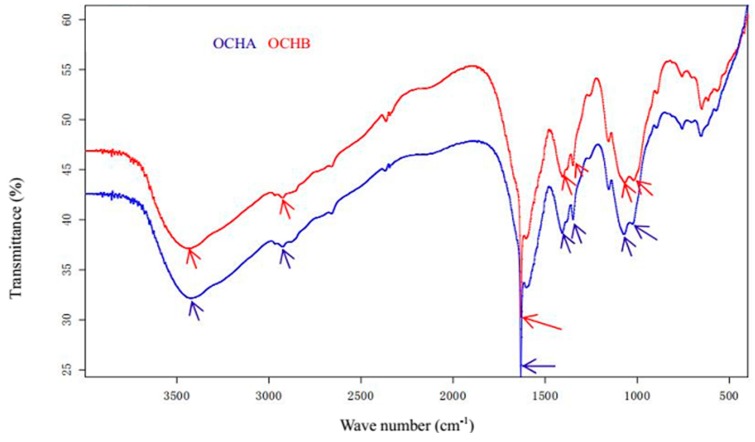
The Fourier transform infrared spectra of OCHA and OCHB. The blue line is the infrared spectrum of OCHA. The red line is infrared spectrum of OCHB.

**Figure 5:**
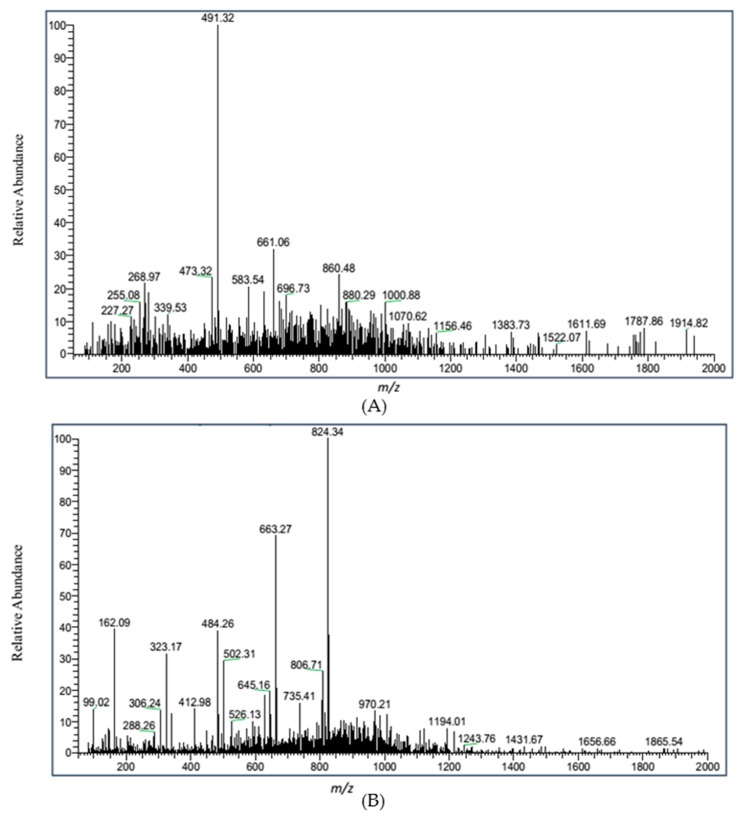
The negative ion electrospray ionization mass spectrometry (ESI-MS) spectra of: OCHA (**A**); and OCHB (**B**). The undelined *m*/*z* numbers might be decuded as some fragment ion peaks.

The FT-IR of OCHA and OCHB were shown in Figure 4, which demonstrated the characteristic absorption peaks of oligochitosan, including those peaks respectively at 3448 cm^−1^ (the absorption of stretching vibration of associated –OH groups), 2932 cm^−1^ (the absorption of stretching vibration of C–H bond), 1632 cm^−1^ (the absorption of flexural vibration of N–H bond), 1410 cm^−1^ (the absorption of flexural vibration of O–H bond), 1340 cm^−1^ (the absorption of stretching vibration of C–N bond), 1070 cm^−1^ (the absorption of stretching vibration of C–O–C bond) and 1020 cm^−1^ (the absorption of stretching vibration of C–OH bond). The ESI-MS spectra of OCHA and OCHB were presented in Figure 5 with the full *m*/*z* range as 50–2000 under negative scanning model. All the above analyses proved the oligochitosan nature of OCHA or OCHB.

### 3.2. Plant and Fungal Materials

The plant materials were the healthy stems of *Z. bungeanum* collected from the plant nursery of Northwest A&F University (Yangling, China). The pathogen of dry rot, *F. sambucinum*, was isolated and preserved in our lab during a previous study [4]. *F. sambucinum* was cultured and preserved on Potato Dextrose Agar (PDA) medium. The aerobic submerged culture was carried out in Potato Dextrose Broth (PDB) at 25 °C with continuous shaking at 150 rpm.

### 3.3. Evaluation of Protective Effects of Oligochitosan on Stems of Z. bungeanum against Dry Rot

The healthy uniform stems of *Z. bungeanum* with the diameter of about 2 cm were collected as the plant materials. The surface of stem was washed by the running water and then cleaned by wet sterile cotton. Six inoculated sites were randomly selected on each stem, all of which were nearly round with the diameter about 5 mm. Each site was punctured for seven tiny stab wounds by the autoclaved dissecting needle. The *F. sambucinum* that was cultured on PDA plates for 7 days was taken as the inoculum, which was punched by the autoclaved hole-puncher with the diameter of 5 mm. Each mycelial plug was placed hyphae side down onto the surface of each inoculated site with stab wound. And then inoculated sites were wrapped by sterile cotton soaked in oligochitosan solutions with different concentrations. Both of OCHA and OCHB solutions were prepared using sterile distilled water and filtered through a sterile filter membrane (pore size, 0.45 µm), the concentrations of which were respectively 0.5 and 1.0 mg/mL. The control was treated with sterile distilled water. Each stem included three replicates of oligochitosan treatment and three controls. Each treatment was applied for twenty stems. Hence, each oligochitosan treatment included 60 inoculated sites, and control treatment included 240 inoculated sites. The morphological lower side of stem with cut was rinsed in sterile water to provide necessary water for the normal metabolism of plant. All the treated stems were placed in the growth chamber at 25 °C under 12 h daily illumination of approximately 2000 lx. After 7 days, it was carried out to observe symptom appearance of dry rot and count the inoculated sites infected successfully by *F. sambucinum* for all stems. The infection incidence (%) was calculated by the percent of infected sites in total inoculated sites, which was taken as the indicator to evaluate the protective effects of oligochitosans on *Z. bungeanum* stems against dry rot. All the data analyzed in the present research were the experimental results for the plants in August 2015. Moreover, preliminary experiments were also carried out for the plants in April and June 2015 and positive results were obtained (data not shown).

### 3.4. Determination of the Effect of Oligochitosan on Growth of F. sambucinum

Inhibitory effects of OCHA and OCHB on the growth of radial colony and submerged biomass of *F. sambucinum* were conducted. Both of OCHA and OCHB were separately dissolved in sterile distilled water, and then filtered through a sterile filter membrane (pore size, 0.45 µm). For the radial colony growth determination, the sterile oligochitosan solution was added into PDA at 60 °C with the final concentrations of 0.2, 0.4, 0.6, 0.8 and 1.0 mg/mL, respectively, which were then mixed rapidly and poured into Petri dishes (diameter, 9 cm). The same volume of sterile distilled water was added into PDA as the control. After the PDA plate solidified, a 5-mm-diameter mycelial plug of *F. sambucinum* was inoculated on the center of the PDA plate and incubated at 25 °C in dark. The diameter (R) of *F. sambucinum* colony was measured on Days 2, 4, 6, 8 and 10, respectively [20]. Each treatment was carried out for three duplicates. Radial growth inhibition (RGI) was calculated according to Equation (1):

RGI (%) = (R_0_ − R) × 100%/R_0_ (1)

where R_0_ is the colony diameter of control, and R is the colony diameter of treatment.

To determinate the effects of oligochitosan on mycelial biomass of *F. sambucinum*, the submerged culture was carried out in PDB liquid medium. Each 1000-mL flask was filled with 500 mL PDB medium, and then sterile oligochitosan solution was added into PDB. The final concentrations of both OCHA and OCHB in PDB were separately 0.2, 0.4, 0.6, 0.8 and 1.0 mg/mL. And then, 0.5 mL seven-day-old suspension culture of *F. sambucinum* in PDB was taken as the inoculum and injected into each flask containing PDB liquid medium with oligochitosan supplementation. All the flasks were maintained on a rotary shaker at 150 rpm at 25 °C. The mycelial biomass was respectively measured on the day of 2, 4, 6, 8 and 10 by filtrating under vacuum to obtain the mycelia, which were further lyophilized to a constant dry weight (DW) and expressed as gram per liter [40]. Each treatment was carried out for three duplicates. The effect of oligochitosan on mycelial biomass of *F. sambucinum* was calculated by the biomass growth inhibition (BGI) according to Equation (2):

BGI (%) = (DW_0_ − DW) × 100%/DW_0_ (2)

where DW_0_ is the mycelial dry weight of control, DW is the mycelial dry weight of treatment.

### 3.5. Assays of PAL, PPO and POD Activities in Z. bungeanum Twigs

In this investigation, the effects of oligochitosan on defense-related enzymes in *Z. bungeanum* were detected. The changes of PAL, PPO and POD activities were taken as the evaluation indicators. The fresh healthy uniform twigs of *Z. bungeanum* in current year were taken as the elicited materials. All twigs were excised at the base of stem and each of them was then promptly placed in 5 mL eppendorf tubes containing 1 mL oligochitosan solution. The gradient concentrations of OCHA and OCHB were separately 0.5 and 1.0 mg/mL. Control twig was treated by sterile distilled water. After all of the oligochitosan solution was absorbed by the plant (about 5–7 h), the twigs under the same treatment were immediately transferred to a 100 mL glass beaker containing 30 mL sterile distilled water. And then all the treated twigs in glass beakers were placed in growth chamber at 25 °C under 12 h daily illumination of approximately 2000 lx. The twigs were respectively harvested at 12, 24, 48, 72 and 96 h after elicitation. Liquid adhering to the surface of twigs was removed by paper. The harvested fresh twigs were immediately used for extraction of crude defense-related enzymes. Each treatment was carried out in triplicate.

The extraction of PAL was conducted according to the method as described in our previous study [41]. The harvested twigs (0.5 g in fresh weight, FW) were cut into small pieces and then homogenized in pre-cooling 5 mL borate buffer saline (BBS) (0.05 mg/mL, pH 8.8), containing 5.0 mmol/L β-mercaptoethanol. The homogenate was then centrifuged at 10,000 rpm for 20 min at 4 °C and then the supernatant was collected as the enzyme extract of PAL.

The determination of PAL activity was calculated by the amount of trans-cinnamic acid, which was the conversion product of l-phenylalanine by PAL catalyzing [42]. The reaction mixture consisted of 100 μL l-phenylalanine (0.02 mol/L), 50 μL enzymatic extract, and 50 μL BBS, which was incubated at 40 °C for 60 min. After then, 50 μL HCl (2 mol/L) was added into the reaction mixture to terminate the reaction. The absorbance at 290 nm of the reaction mixture was recorded by micro-plate spectrophotometer. One unit (U) of PAL activity is defined as a change of 0.01 OD at 290 nm per minute per gram fresh weight. The results were presented as U·min^−1^·g^−1^ FW.

The crude enzymes of PPO and POD were extracted using the same methods as described in our previous research [41]. The treated twigs of *Z. bungeanum* (0.5 g, FW) were cut into small pieces and then ground in pre-cooling 5 mL phosphate buffer saline (PBS) (0.05 mol/L, pH 6.8) containing 1% polyvinyl polypyrrolidone (PVPP). The homogenate was centrifuged at 10,000 rpm for 20 min at 4 °C and the supernatant was used for the determination of PPO or POD activities.

The reaction mixture for PPO assay contained 50 μL of enzymatic solution, 100 μL of 0.05 mol/L catechol and 50 μL PBS (0.05 mol/L, pH 8.8), and it was monitored by measuring the change of absorbance at 398 nm for 2 min. One unit (U) of PPO activity is defined as a change of 0.01 OD at 398 nm per minute per gram fresh weight. The results were presented as U·min^−1^·g^−1^ FW.

POD activity assay was conducted by the reaction of 10 μL crude enzymatic solution, 25 μL guaiacol (1%), 25 μL H_2_O_2_ (1%) and 150 μL PBS (0.05 mol/L, pH 8.8). After the reaction mixture was incubated for 10 min at 37 °C, its absorbance at 470 nm was recorded with a micro-plate spectrophotometer. One unit (U) of PPO activity is defined as a change of 0.01 OD at 470 nm per minute per gram fresh weight. The results were presented as U·min^−1^·g^−1^ FW.

### 3.6. Detection of Total Phenolics Changes in Z. bungeanum Twigs

It has been widely reported that phenolic compound is one of the numerous antimicrobial compounds in plant tissue [43]. It has been reported that there were phenolic compounds existing in *Z. bungeanum,* such as flavonoids [44]. To investigate the effect of oligochitosan on the accumulation of total phenolics, the twigs of *Z. bungeanum* were treated with oligochitosan as described in Section 3.5. The gradient concentrations of OCHA and OCHB were separately 0.5 and 1.0 mg/mL. Control twig was treated by sterile distilled water. The twigs were collected respectively on Days 2, 4, 6, 8 and 10 after treatment as the plant materials for extraction of total phenolics.

The extraction of total phenolics in twigs was carried out by ethanol-extraction method according to the previous research with some modification [45]. The treated twig of *Z. bungeanum* (0.5 g, FW) was cut into small pieces and then ground in 5 mL 60% ethonal as homogenate, which was then subjected to ultrasonic treatment for 30 min. After that, the homogenate was centrifuged at 10,000 rpm for 20 min at 4 °C, and the supernatant was collected as the crude extract of total phenolics.

The content of total phenolics was measured by Folin-Ciocalteu method with gallic acid as a standard by micro-plate spectrophotometry [43,46]. The reaction mixture contained 50 μL sample and 100 μL Folin-Ciocalteun reagent (1N), which was shaken adequately and then kept stable for 5 min. After that, 100 μL Na_2_CO_3_ solution (10%) was added into the reaction mixture, mixed well and incubated at 25 °C for 2 h. The absorbance of reaction mixture was measured at 765 nm. The phenolic contents were calculated employing the standard curve established with gallic acid. The total phenolic content was expressed as gallic acid equivalent (GAE) in milligrams per gram fresh weight (FW), which was presented as GAE·mg·g^−1^ FW [19].

### 3.7. Statistical Analysis

The experiment was conducted twice with three duplicates of each treatment. As the results of the two experiments were very similar, the data were pooled before analysis. All of the results were represented by their mean values and the standard deviations (SD). The experimental data were submitted to analysis of variance to detect significant differences by PROC ANOVA of SAS version 8.2 (SAS Institute Inc., Cary, NC, USA).

## 4. Conclusions

As shown in the present study, two oligochitosans, i.e., OCHA and OCHB, displayed significant protective effects on *Z. bungeanum* stems against dry rot, inhibiting the growth of *F. sambucinum* in vitro directly, and induced defensive responses in Z. bungeanum remarkably. Various effects of OCHA and OCHB might be associated with their differences in components, molecular weight, the degree of acetylation and polymerization, and preparation methods. Prominent efficacy of oligochitosan on control of dry rot of *Z.* *bungeanum* stems means it is promising to use oligochitosan as a natural fungicide to partially substitute for utilization of synthetic fungicide. This is, to the authors’ knowledge, the first report on the protective function and biochemical mechanisms of oligochitosan for *Z. bungeanum* stems against dry rot. However, further research is worth investigating on molecular mechanism of oligochitosan against fungal pathogens, as well as the specific relationship between structure and function, which would establish the basement for appropriate application of oligochitosan for integrated management of plant disease.

## Figures and Tables

**Table 1:** Effects of oligochitosan A (OCHA) and oligochitosan B (OCHB) on the radial colony and biomass growth of *F. sambucinum*.

Oligochitosan Concentration (mg/mL)	Detection Time (day)	RGI (%)		BGI (%)	
		OCHA	OCHB	OCHA	OCHB
0.2	2	26.78 ± 1.13 y	26.47 ± 1.01 y	37.11 ± 4.27 t	42.37 ± 6.11 r
	4	32.14 ± 1.27 x	39.06 ± 3.36 v	39.74 ± 3.36 s	44.12 ± 4.08 q
	6	40.64 ± 1.58 uv	32.70 ± 1.82 x	34.84 ± 1.91 u	39.93 ± 5.10 s
	8	42.74 ± 1.77 t	35.18 ± 2.31 w	31.63 ± 3.60 v	38.30 ± 3.97 st
	10	42.65 ± 1.08 t	36.06 ± 1.42 w	25.33 ± 2.37 w	25.21 ± 1.87 w
0.4	2	41.83 ± 1.13 tu	36.49 ± 1.70 w	54.19 ± 2.59 mn	59.15 ± 4.48 kl
	4	55.51 ± 0.76 p	53.74 ± 2.20 q	53.47 ± 3.44 n	60.81 ± 6.89 k
	6	51.10 ± 3.15 qr	46.37 ± 3.15 s	55.61 ± 2.24 m	63.98 ± 6.19 j
	8	40.07 ± 2.31 uv	43.06 ± 2.91 t	50.12 ± 2.24 o	58.75 ± 4.21 l
	10	39.17 ± 1.42 v	41.89 ± 3.88 tu	49.03 ± 1.87 op	48.18 ± 3.58 p
0.6	2	51.00 ± 1.70 r	56.71 ± 3.44 p	60.84 ± 5.95 k	64.78 ± 5.95 j
	4	64.02 ± 1.27 lm	66.25 ± 2.26 k	63.27 ± 3.00 j	67.65 ± 5.19 i
	6	65.25 ± 1.82 kl	72.03 ± 0.00 g	66.90 ± 5.30 i	70.52 ± 1.27 h
	8	68.10 ± 1.34 j	75.59 ± 1.92 f	63.50 ± 3.25 j	77.05 ± 3.05 f
	10	71.55 ± 1.08 gh	78.38 ± 1.08 d	58.67 ± 1.64 l	74.18 ± 2.18 g
0.8	2	61.78 ± 0.92 no	60.63 ± 3.96 o	67.45 ± 5.18 i	71.49 ± 6.11 h
	4	69.60 ± 0.76 ij	69.16 ± 1.02 ij	71.18 ± 4.93 h	75.95 ± 3.00 f
	6	71.56 ± 1.11 gh	75.76 ± 1.27 ef	74.09 ± 1.95 g	81.37 ± 3.21 d
	8	75.52 ± 3.18 f	82.47 ± 0.67 c	80.95 ± 2.72 d	85.53 ± 2.20 c
	10	80.95 ± 1.52 c	88.86 ± 4.27 b	85.94 ± 3.30 c	88.82 ± 1.09 b
1.0	2	62.78 ± 1.23 mn	65.35 ± 4.08 kl	70.23 ± 5.18 h	76.84 ± 6.42 f
	4	70.04 ± 0.76 hi	73.19 ± 2.31 g	75.62 ± 2.04 fg	79.14 ± 5.99 e
	6	77.41 ± 3.80 de	80.76 ± 2.45 c	80.93 ± 2.41 d	86.22 ± 2.57 c
	8	82.41 ± 3.68 c	88.85 ± 1.57 b	81.95 ± 2.58 d	88.78 ± 2.24 b
	10	89.73 ± 1.86 b	95.69 ± 1.08 a	84.64 ± 1.82 c	92.34 ± 2.07 a

Each value was expressed as mean ± standard deviation (*n* = 3). RGI and BGI were two indicators to evaluate the inhibitory effects of oligochitosan on radial colony growth and biomass growth of *F.* *sambucinum*. The significant difference analyses of RGI and BGI were carried out at *p* = 0.05 level. Different letters (i.e., a–y in the columns of OCHA and OCHB) indicated significant differences among the treatments.

## Abbreviations

OCHA	Oligochitosan A
OCHB	Oligochitosan B
*M*w	Molecular weight
DA	Degree of acetylation
DP	Degree of polymerization
PAL	Phenylalanine Ammonia Lyase
PPO	Polyphenoloxidase
POD	Pperoxidase
RGI	Radial Growth Inhibition
BGI	Biomass Growth Inhibition
